# From concept to measurement: initial validation of the Ultra-Processed Food Consumption Scale

**DOI:** 10.1007/s40519-026-01825-9

**Published:** 2026-02-20

**Authors:** Michail Mantzios, Misba Hussain, Kyriaki Giannou

**Affiliations:** 1 Department of Psychology, School of Health and Life Sciences, Birmingham City University, Birmingham, UK; 2 Department of Psychology, Faculty of Health and Life Sciences, De Montfort University, Leicester, UK

**Keywords:** Ultra-Processed Food Consumption Scale, Ultra-processed foods, NOVA classification, Food addiction, Mediterranean diet

## Abstract

**Purpose:**

High consumption of ultra-processed food (UPF) has been consistently linked to negative health outcomes, but existing tools for assessing UPF consumption are often lengthy, culturally specific, or psychometrically underdeveloped. The present research aimed to develop and validate the Ultra-Processed Food Consumption Scale (UPFCS); that is, a brief, psychometrically sound, and cross-culturally applicable measurement.

**Methods:**

In Study 1 (*n* = 325), exploratory factor analysis (EFA) was conducted to examine the factorial structure and internal consistency of the 30-item UPFCS. Study 2 (*n* = 129) evaluated test–retest reliability across a 2-week interval, and Study 3 (*n* = 144) assessed convergent validity by examining associations to Mediterranean diet adherence, body mass index (BMI), and food addiction, as well as testing the moderating role of Mediterranean diet adherence.

**Results:**

EFA and internal consistency supported a unidimensional structure, while test–retest reliability was moderate to strong. UPFCS scores were positively associated with food addiction symptoms, but not with BMI or Mediterranean diet adherence. Exploratory moderation analysis indicated that adherence to a Mediterranean diet attenuated the relationship between UPF consumption and food addiction symptoms, with the effect strongest among those with low Mediterranean dietary adherence.

**Conclusions:**

The UPFCS demonstrates robust psychometric properties and practical utility for assessing ultra-processed food consumption in behavioural and nutritional research.

**Supplementary Information:**

The online version contains supplementary material available at 10.1007/s40519-026-01825-9.

## Introduction

A growing body of epidemiological and clinical evidence links high UPF intake to a wide range of adverse health outcomes [2, 4, 11], making the monitoring and measurement of ultra-processed food consumption essential for research and practice purposes. Systematic reviews and meta-analyses have consistently demonstrated that elevated consumption of UPF is associated with increased risks of all-cause mortality, cardiovascular disease, type 2 diabetes, obesity and cancer diagnoses [20, 22, 34]. Moreover, UPFs have been implicated in mental health difficulties [19, 21]. Therefore, findings collectively highlight the urgent need for scalable, reliable, and universally applicable tools to assess UPF consumption to advance behavioural science research aimed at reducing the burden of diet-related diseases.

The exponential growth in research on food processing is closely aligned with the NOVA classification system, which has become the dominant framework for categorising foods from unprocessed to ultra-processed [26, 27]. Despite its widespread adoption, the methods used to evaluate UPF intake remain somewhat unstandardised, lacking the possibility for replications and comparisons and making their use in behavioural science research impractical. Instruments such as the Food Frequency Questionnaire (FFQ), including NOVA-adapted and country-specific adaptations, alongside tools like the NOVA-27 UPF Screener, 24-h dietary recalls, and 7-day dietary records, have been employed with varying degrees of success [1, 10, 13, 14, 28, 29, 31]. However, these methods are frequently criticised for their length, complexity, and resource-intensive nature, as well as the lack of validation studies [13, 24], limiting their scalability and cross-cultural applicability, despite the recognition of country-specific instruments carrying a unique value and having a place in the research spectrum of UPF intake.

## Present research

In response, the present research introduces a scale that addresses the pressing need for a psychometrically sound and low-burden instrument that can be easily administered across diverse populations; that is, the Ultra-Processed Food Consumption Scale (UPFCS). To standardise the scale, three studies were conducted. Study 1 aimed to establish the factorial structure using Exploratory Factor Analysis (EFA) and internal consistency utilising alpha and omega coefficients. Study 2 aimed to determine the scale’s stability over time through test–retest reliability. Finally, Study 3 aimed to confirm the scale’s construct validity by correlating UPFCS scores with measures of food addiction and Mediterranean diet adherence, as food addiction was highlighted in the literature on the association to UPF (e.g., [12, 35]) , and Mediterranean dieting as being the opposite to highly processed foods.

## Study 1

### Method

#### Participants

The sample comprised 325 participants, with a mean age of 40.98 years (SD = 12.85) and a mean BMI of 27.25 (SD = 5.51). The gender distribution included 166 males and 158 females. Ethnic background was as follows: White British (*n* = 271), Black British (*n* = 26), British Asian (*n* = 15), Mixed British (*n* = 6), and 7 participants selected “other” without sharing any further details.

### Measures

#### Ultra-Processed Food Consumption Scale development

**Fig. 1:**
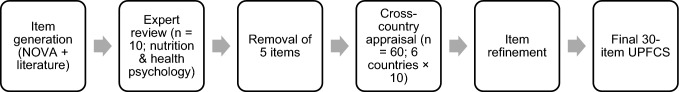
Flow diagram: UPFCS development

Prior to Study 1, a structured multi-phase process (i.e. item generation → expert review → cross-country item appraisal → item refinement; see Fig. 1) was initiated, and is described in detail below:

1. Item generation: initially, an item pool of 35 items was generated based on the NOVA classification system, with considerations of additional literature (e.g., [3, 8, 26]) for ultra-processed foods that included industrial formulations with ingredients not found in home cooking (e.g., emulsifiers, artificial sweeteners, etc.) to be included in the initial version of the scale, and with a focus on capturing UPF intake cross-nationally. Four criteria were used to select and generate items: (a) NOVA marker ingredients/formulation indicative of industrial additives (e.g., instant noodles); (b) cross-national relevance indicative when item was present in more than 5 countries, or a cultural adaptable alternative was part of the item (e.g., sweetened milk teas/soft drinks); (c) ease of respondent recognition indicative in common language used and recognised by consumers (e.g., frozen pizza), and finally; (d) low ambiguity indicative that ultra and minimally processed foods would be distinct (e.g., Packaged cereal—sweetened types). Several rounds of assessments, discussions and final agreements led to the final 35 items.
2. Expert reviewers: ten experts in nutrition (*n* = 5) and health psychology (*n* = 5) reviewed the items to establish initial content validity, ensuring alignment with theoretical and empirical definitions of ultra-processed foods. Experts (10 female, geographic location: USA *n* = 2, Greece *n* = 1, UK *n* = 2, China *n* = 2, Mexico *n* = 1, Japan *n* = 1, Turkey *n* = 1) were purposefully invited based on professional qualifications and >5 years experience in dietary research and/or clinical experience. Experts received the 35-item pool and were asked to complete relevance to UPFs and cultural applicability by judging each item on a 3-point scale (to retain, revise or remove) following protocols for a single-round structured review [9]. The five items considered by five or more experts that should be removed due to interference with cultural comparisons, or controversial in representing UPFs, were: (1) Yakult (7 remove), (2) granola bars (8 remove regardless of suggested as being an independent item, or as an item included in packaged cereal item), (3) bento box (item was noted that it matched item 17 of the final version—6 remove), (4) microwave curries (6 remove), and (5) soy sauce additives/oyster sauce/curry pastes (8 remove), meanwhile, open-text responses allowed for items to be consolidated, item refinement or removed, based on the written feedback. The remaining 30 items had a minimum of 90% agreement on retaining them, and qualitative comments provided insight into why revisions may have been suggested by experts (e.g., including culturally relevant food examples to support participant understanding). The authorship team judged that there was no case for returning the final version to the experts for a second round as only minor revisions were proposed and these were incorporated into the final version.
3. Cross-country appraisal: following refinement and removal of 5 items, the scale underwent participant appraisals using a convenience sample (*n* = 60, 30 female) from varied ethnic backgrounds and good command of English (*n* = 10 for each of the following countries: UK, USA, France, Mexico, South Korea, and Japan) to ensure presence and relevance within each country and cross-cultural applicability. Participants were given an option for each item “N/A—I never consume those foods”, in addition to their consumption frequency for each item using the five-point Likert described below. Participants from all countries did not select the “N/A” option for any item and reported consumption of foods listed in the final 30-item version.
4. The final 30-item UPFCS was reassessed for the potential assessment of bias towards Western perceptions of UPFC. A comparison between Asian and Western countries, for both expert reviews and cross-country appraisals, led to non-significant differences. The data did not indicate any patterns that were concerning on the cross-cultural applicability of future research. A final round of reviewing items for clarity led to no further changes. The complete 30-item UPFCS is provided in Supplementary Material (Appendix A/Table S1), and the description of the scale is presented below.

*The Ultra-Processed Food Consumption Scale (UPFCS)*

The UPFCS is a 30-item self-report instrument (provided in Supplementary Material, Table S1) designed to quickly and reliably capture the frequency of consuming various ultra-processed foods (UPFs). Respondents rate their consumption frequency for each item using a five-point Likert scale from 0 (Never or less than once a month) to 4 (Daily or more). Sample items make reference to instant meals and processed meats. Final scores are calculated by summing the responses to yield a total score between 0 and 120, where higher scores indicate a greater intake of ultra-processed foods.

### Procedure

Participants were recruited through an online platform (i.e. Prolific), which provided the ability for them to be reimbursed for their participation time (£6.00/h). Participants were provided with a link to fill in the survey through an online platform (i.e. QuestionPro), and first viewed the participant information sheet and consent form, and upon consenting, were presented with the demographics page and the UPFCS. After completing the scale, the participants were directed to a debriefing page before their participation ended.

### Analysis

To ensure data fitness, data screening was conducted by evaluating the presence of outliers, multivariate normality, linearity, and homogeneity of variance. Kaiser–Meyer–Olkin (KMO) for sampling adequacy and Bartlett’s test for sphericity were also assessed before proceeding with any attempts to conduct inferential statistics. Once all assumptions were satisfied, exploratory factor analysis (EFA), with principal axis factor extraction and oblique rotation, was performed. Criteria to evaluate factor extraction were screeplot evaluation, eigenvalues (>1), and item loading greater than 0.40 [17, 33].

### Results

An exploratory factor analysis (EFA) using principal component analysis was conducted on the 30 items assessing frequency of ultra-processed food (UPF) consumption. The suitability of the data for factor analysis was confirmed by the Kaiser–Meyer–Olkin (KMO) measure of sampling adequacy, which was 0.882, indicating meritorious sampling adequacy. Bartlett’s test of sphericity was significant, *χ*^*2*^(435) = 2354.16, *p* < 0.001, confirming that correlations among items were sufficiently large for EFA.

Although the initial eigenvalues greater than 1 suggested a seven-factor solution, inspection of the scree plot revealed a distinct inflection after the first component, indicating that a single-factor solution provided the only interpretable structure. The first component accounted for 31.65% of the total variance (eigenvalue = 9.50), while the second and subsequent components each explained less than 7% of additional variance.

All items loaded positively on the first factor, with loadings ranging from 0.45 to 0.75, further suggesting that the items reflected a single underlying construct. Communalities ranged from 0.42 to 0.75, indicating that each item contributed meaningfully to the latent factor (see Table 1). The single factor represents a coherent dimension reflecting ultra-processed food consumption.

**Table 1:** Item loadings and communality

Item	Loading	Communality
1. Sweetened milk teas	0.55	0.55
2. Pre-packaged iced teas	0.54	0.55
3. Flavoured/powdered milk drinks	0.70	0.69
4. Fruit drinks with added sugar	0.61	0.53
5. Potato chips, shrimp crackers	0.60	0.68
6. Packaged cookies, cakes	0.48	0.75
7. Instant or cup noodles	0.58	0.71
8. Sweetened/flavoured drinks	0.60	0.64
9. Packaged cereal	0.45	0.56
10. Packaged sweet buns/pastries	0.51	0.58
11. Pre-packaged croissants	0.58	0.69
12. Frozen waffles or pancakes	0.75	0.64
13. Fish balls, luncheon meats	0.66	0.66
14. Sausages, deli meats	0.54	0.54
15. Canned meat spreads	0.72	0.62
16. Frozen pizzas or lasagne	0.52	0.42
17. Instant rice bowls	0.66	0.68
18. Congee mixes, instant soups	0.59	0.61
19. Flavoured instant oatmeal	0.69	0.55
20. Toaster pastries/pancakes	0.71	0.59
21. Packaged salad dressings	0.42	0.60
22. Instant gravy powders	0.51	0.51
23. Sweetened spreads (e.g., jam, chocolate)	0.67	0.55
24. Bubble tea with toppings	0.60	0.60
25. Artificial whipped cream	0.61	0.53
26. Jelly cups, instant puddings	0.45	0.53
27. Sweetened soy or coconut milk	0.69	0.68
28. Processed cheese slices	0.51	0.46
29. Ready-to-drink protein shakes	0.53	0.62
30. Plant-based burgers/nuggets	0.48	0.58

Internal consistency for the 30-item scale was excellent, with Cronbach’s *α* = 0.91, and McDonald’s *ω* = 0.91, further supporting the unidimensionality of the scale. Collectively, these results indicate that the UPFCS is a psychometrically sound and internally consistent unidimensional measure.

## Study 2

### Method

#### Participants

The sample comprised 129 participants, with a mean age of 42.09 years (SD = 10.34) and a mean BMI of 27.03 (SD = 5.48). The gender distribution included 63 males and 65 females. The ethnic distribution was as follows: White British (*n* = 103), White Other *(n* = 5), Black British (*n* = 11), Mixed Race (*n* = 3), Chinese British (*n* = 3), and Pakistani British (*n* = 3).

### Measures

*Ultra-Processed Food Consumption Scale* (*UPFCS*). See Study 1 for description. Cronbach’s alpha values for Study 2 were 0.93 for baseline test and 0.92 for retest.

### Procedure

As in Study 1, participants were invited through Prolific and the research was administered through Question Pro. Participants were asked to complete a food and perception questionnaire twice, over 2 weeks. In both instances, participants were provided with a link, where they viewed the participant information and consent forms, and once they consented, proceeded to the demographics page and the UPFCS. Once they completed the UPFCS, participants were directed to a debriefing page, which concluded their participation.

### Analysis

Test–retest reliability was assessed to show the reliability over time of the UPFCS. Pearson’s correlation was used with strong correlation coefficients estimated at a value above *r* = 0.50 [6]. In addition to the total score Pearson correlation, we computed item-level test–retest correlations to identify items with low temporal stability. All data were analysed using IBM SPSS 28.

### Results

Test–retest correlations demonstrated a moderate level of stability over time. The original measure correlated significantly, but moderately, with the retest scores, *r* = 0.487, *p* < 0.001. Item-level Pearson’s correlations between baseline and 2-week retest ranged from 0.21 to 0.65. Intraclass correlation coefficients (two-way mixed, absolute agreement) ranged from 0.23 to 0.64, with only items 2, 3, 4, 6 and 8 not demonstrating poor stability according to established ICC benchmarks (i.e. ICC < 0.50; see [18]). Cicchetti’s [5] more lenient criteria with values between 0.40 and 0.59 to be considered “Fair” would qualify more items to be within an acceptable range, but still the item-level analyses are indicative of further research being required on temporal stability. Then again, Hemphill [16] suggested that in behavioural science, and in this case consumption frequency would fall under this category, correlation of *r* = 0.3 and 0.5 could be considered “medium” and "large", respectively. Despite different guidance and criteria, findings are further evaluated in the discussion of this manuscript, and a detailed output can be found in the Supplementary materials (see Table S2).

## Study 3

### Method

#### Participants

The sample comprised 144 participants, with a mean BMI of 26.58 (SD = 5.46). The gender distribution included 65 males and 75 females. Participants reported a wide range of ethnic backgrounds, with the majority identifying as White British (*n* = 84), followed by White Other (*n* = 13), Black British (*n* = 13), Asian British (*n* = 9), Mixed (*n* = 5), and Other/Unspecified (*n* = 20).

### Measures

*Ultra-Processed Food Consumption Scale* (*UPFCS*; [25]). See Study 1 for description. Cronbach’s Alpha for Study 3 was 0.93.

*Mediterranean Diet Adherence Questionnaire* (*MD*; [23]). The MD assesses adherence to a Mediterranean diet, and consists of 9 items. Sample items reference legumes, fish and meat products. A score of 1 was assigned when the participant responded to the predetermined quantity proposed in the validation research (e.g., greater and equal to 1 serving/day) for fruit and vegetable, while 0 if the threshold was not met. Total scores could range from 0 to 9, with higher scores indicating greater adherence. Cronbach’s Alpha was 0.908.

*Modified Yale Food Addiction Scale—Version 2.0* (*mYFAS 2.0*; [32]). The mYFAS 2.0 proposes a measurement of addictive eating behaviour. The scale is composed of 13 items, rated on an eight-point Likert scale (from 0 = never to 7 = every day) assessing addictive eating behaviours (e.g., “I had such strong urges to eat certain foods that I could not think of anything else”; “I ate to the point where I felt physically ill”). The mYFAS 2.0 was scored on the basis of a symptom count where scores could range from 0 to 11. Cronbach’s Alpha was 0.918.

### Procedure

Participants were recruited from Prolific and were reimbursed for their participation time (£6.00/h) as in the previous studies. Participants were provided with a link to fill in the survey through QuestionPro, and first viewed the participant information sheet, the consent form, and, upon consenting, were presented with the demographics page and the scales listed above. After completing the scales, the participants were directed to a debriefing page before their participation ended.

### Analysis

Pearson correlation analyses were conducted to examine the relationships among the Ultra-Processed Food Consumption Scale (UPFCS), Mediterranean dieting (MD), body mass index (BMI), and food addiction symptoms as measured by the Yale Food Addiction Scale (YFAS). A moderation analysis was conducted using PROCESS Model 1 [15] to examine whether the relationship between UPFCS and food addiction symptoms (YFAS scores) was moderated by adherence to a MD. The MD score was split into three groups (low, medium, high adherence) using sample tertiles. Group differences in UPFCS and YFAS scores were tested using one-way ANOVA, and post hoc pairwise comparisons used Bonferroni correction.

### Results

A significant positive correlation was found between UPFCS and YFAS scores, *r* = 0.27, *p* = 0.001, indicating that higher consumption of ultra-processed foods was associated with greater food addiction symptoms. Similarly, BMI was significantly positively correlated with YFAS scores, *r* = 0.33, *p* < 0.001, suggesting that individuals with higher BMI reported more severe food addiction symptoms (Table 2).

**Table 2:** Pearson’s correlation between UPFC, MD, BMI and YFAS

	UPFCS	MD	BMI
UPFCS	–		
MD	0.018	–	
BMI	−0.033	0.011	–
YFAS	0.270**	−0.124	0.328**

*UPFCS* Ultra-processed Food Consumption Scale, *MD* Mediterranean dieting, *BMI* body mass index, *YFAS* Yale Food Addiction Scale

The moderation analysis examining whether the relationship between Ultra-Processed Food Consumption (UPFC) and food addiction symptoms (YFAS scores) was moderated by adherence to a Mediterranean diet (MD) showed an interaction between UPFC and MD that approached significance (*b* = −0.0390, *p* = 0.0999), suggesting a potential moderating effect.

To probe the interaction, conditional effects of UPFCS on YFAS were examined at three levels of MD (16th, 50th, and 84th percentiles). The effect of UPFCS on YFAS was significant at low levels of MD (*b* = 0.2022, *p* = 0.0003), but not at moderate (*b* = 0.0853, *p* = 0.1526) or high levels (*b* = 0.0463, *p* = 0.5485). This pattern suggests that the positive association between UPFCS and food addiction symptoms is stronger among individuals with lower adherence to the Mediterranean diet (Table 3).

**Table 3:** Conditional effects of UPFCS on YFAS at values of Mediterranean dieting (MD)

MD (percentile)	Beta	SE	*t*	*p*	95% CI (LLCI, ULCI)
−1SD	0.202	0.054	3.74	0.0003	[0.0953, 0.3091]
Moderate	0.085	0.059	1.44	0.1526	[−0.0319, 0.2025]
+1SD	0.046	0.077	0.60	0.5485	[−0.1059, 0.1985]

## Discussion

The studies in the present manuscript introduce an initial validation of the Ultra-Processed Food Consumption Scale (UPFCS), a novel measure designed to capture Ultra-Processed Food (UPF) intake. The findings across three studies provide strong preliminary evidence for the scale’s reliability, validity, and applicability across diverse samples Study 1 demonstrated that the UPFCS is best represented as a unidimensional construct, reflecting the frequency of ultra-processed food consumption. The scale’s high internal consistency, one dimensional loadings and satisfactory communalities indicate that the items coherently represent the intended domain of ultra-processed food consumption. Together, these findings establish the UPFCS as a statistically robust tool for measuring UPF consumption.

Study 2 provided evidence of temporal stability, with a significant test–retest correlation over a 2-week interval; a finding rather important for both longitudinal and intervention research, as well as assessments of policy impact across time. However, findings were not as straightforward when exploring item-level stability over time. Only a handful of items proposed stability over time when following conservative criteria based within clinical and biomedical contexts [18], rather than behavioural self-report items, where most items are acceptable when following more scientifically aligned and fair criteria [5, 16]. Still, the scale may be highly sensitive to external or environmental changes, which would alternatively explain the results. Utilising the UPFCS in an intervention for healthy eating, and observing a significant drop, compared to a control group, would add a proof of concept in determining scale sensitivity, and behavioural change over random error.

Study 3 examined the UPFCS in relation to established diet-related constructs. As expected, higher UPF consumption was associated with greater food addiction symptoms, consistent with existing literature linking UPFs to compulsive eating, overconsumption and food addictive markers [12]. This finding supported the convergent validity of the UPFCS, but interestingly, no significant correlations were found between UPF consumption, BMI, and adherence to Mediterranean dieting. This may reflect the multifactorial nature of BMI and the potential for individuals to consume both healthy and ultra-processed foods simultaneously, observations of processed protein consumption that is on the rise, or potential implications for vegan and vegetarian populations that are consuming UPF when they also fulfil a large portion of Mediterranean dieting. A note to make here is that the lines may be blurred in some cases as to what constitutes healthy eating, despite the processing, and that may suggest that Mediterranean diet adherence and UPFC intake may not be perfectly opposing constructs as we would naturally assume. Importantly, adherence to a Mediterranean diet moderated the association between UPF consumption and food addiction symptoms: the relationship was strongest among individuals with low adherence, suggesting that nutrient-dense dietary patterns may buffer against the addictive potential of UPFs. This finding aligns with historical and evolving evidence on the protective effects of Mediterranean dieting by introducing another dimension on dietary quality and associations to psychological health (e.g., [7]).

Despite its strengths, several limitations should be acknowledged. First, all samples for the studies were recruited online and limited to UK-based participants, restricting generalisability. Second, self-reported dietary measures are inherently subject to recall and social desirability biases. Third, despite the steps taken to enhance cross-cultural applicability during the development, the validation studies occurred at a national level. Fourth, demographic characteristics such as household income, educational attainment, and deprivation indices were not collected in the present studies, and should be included in future research to assess correlations between socioeconomic status and UPF consumption. It may provide valuable insights into UPFC and frequency differences, especially amongst populations for whom UPFs are chosen out of economic necessity. Finally, Items 2, 3, 4, 6, 7, 8, 13, 15, 21, 27, and 28 were the only items that proposed Intraclass Correlation Coefficient greater than 0.4, and represent a more stable representation of the scale over time, which future research needs to consider. Fluctuations may be an outcome of week-to-week availability, pay cycles, or dieting attempts, as well as recall problems and/or social desirability, which need to be considered and controlled for in future research. Despite this limitation, Nunnally [30] argued that statistical criteria should not outweigh content validity, and therefore, we recommend retaining all the items until there is sufficient evidence to support an equivalent shorter form.

Apart from enhancing demographic explorations, future research should examine criterion validity using UPFCS scores with objective dietary intake data (e.g., 24-h recalls) or biomarker-based assessments. Furthermore, cross-cultural translations and measurements testing across languages and regions will enhance the cross-cultural applicability of the scale, while longitudinal data will support findings on obesity and weight gain, as well as disordered eating and physiological outcomes such as cardiometabolic instances. Last, exploring the sensitivity to change of the UPFCS due to interventions, public health campaigns, or psychoeducational material, may offer further directions of surveying public health and behaviour change.

In conclusion, the Ultra-Processed Food Consumption Scale (UPFCS) represents a significant development in dietary assessment of UPF intake. Preliminary data offer a reliable, valid and efficient tool for quantifying UPF intake. Given the global rise in ultra-processed food consumption and its documented health consequences, the UPFCS holds considerable promise for informing research, policy, and intervention strategies aimed at improving dietary health and reducing the burden of diet-related diseases.

## Strengths and limits

A key strength is the strong internal consistency, temporal stability, and convergent validity of the UPFCS across diverse UK samples. The development with expert review and cross-cultural item appraisal further supports practical utility as a low-burden measurement tool for ultra-processed food consumption. The reliance on self-reported data and UK-based samples limits generalisability, highlighting the need for international replication.

### What is already known on this subject?

High consumption of ultra-processed foods is consistently associated with adverse physical and mental health outcomes. The NOVA classification system has become the dominant framework for defining and studying ultra-processed foods in nutritional research. However, existing methods for assessing UPF intake are often lengthy, resource-intensive, culturally specific, restricting their use in behavioural sciences, large-scale research, as well as international comparison studies.

### What this study adds?

This study introduces the Ultra-Processed Food Consumption Scale (UPFCS), a brief, psychometrically robust, and theoretically grounded measure of UPF intake. The findings provide initial evidence that UPF consumption, as measured by the UPFCS, is positively associated with food addiction symptoms, and that adherence to a Mediterranean diet may moderate this relationship.

## Supplementary Information

Below is the link to the electronic supplementary material.

Supplementary file 1.

## Data Availability

Data will be available on request from the corresponding author.
