# Supplementary material for: From concept to measurement: initial validation of the Ultra-Processed Food Consumption Scale
Source: Eat Weight Disord. 2026 Feb 20;31(1):22. doi: 10.1007/s40519-026-01825-9 (PMC13031252; doi:10.1007/s40519-026-01825-9)
Supplement: Supplementary file 1 [file 40519_2026_1825_MOESM1_ESM.docx]

**Table S1. Ultra-Processed Food Consumption Scale (UPFCS; Mantzios, Hussain & Giannou, 2026)**

**Instructions:** Select how often you consume the following items using the provided response options:

- 0 = Never or less than once a month
- 1 = 1–3 times per month
- 2 = 1–2 times per week
- 3 = 3–6 times per week
- 4 = Daily or more

1. Sweetened milk teas or regular sodas/soft drinks
2. Pre-packaged iced teas or sweetened coffees
3. Flavored or powdered milk drinks
4. Fruit drinks with added sugars (e.g., fruit punches, packaged mango juice)
5. Shrimp crackers, flavored rice cakes, packaged mochi, or potato chips
6. Packaged cookies, cakes, or pastries
7. Instant or cup noodles
8. Sweetened or flavored popcorn
9. Packaged cereal *(sweetened/flavored types)*
10. Packaged sweet buns, pineapple cakes, custard bread, or industrial sliced bread
11. Pre-packaged croissants, muffins, or doughnuts
12. Frozen waffles or pancakes
13. Fish balls, luncheon meat, pork floss, or hot dogs
14. Sausages, deli meats, chicken nuggets, fish sticks, or surimi
15. Canned meat spreads or potted meats
16. Frozen pizzas, lasagnas, or canned soups
17. Instant rice bowls or noodle bowls
18. Congee mixes, instant rice porridges, or sugary breakfast cereals
19. Flavored instant oatmeal packets
20. Toaster pastries or packaged scones
21. Packaged salad dressings or commercial mayonnaise/ketchup
22. Instant gravy powders or bouillon cubes
23. Sweetened spreads (e.g., chocolate spreads)
24. Bubble tea with toppings, matcha-flavored desserts, or packaged ice creams
25. Artificial whipped creams or dessert toppings
26. Jelly cups, instant puddings, or candy bars
27. Sweetened soy or coconut milk drinks, or flavored/sweetened dairy milks
28. Processed cheese slices or spreads
29. Ready-to-drink protein shakes
30. Mock meats, packaged tofu desserts, or plant-based burgers/nuggets

**Scoring Guide:**

Total points 0-120 for the whole scale. Higher scores indicate higher consumption of ultra-processed foods.

**Table S2. Item-level test–retest Pearsons correlations and Intraclass Correlation Coefficient (ICC).**

| **Item** | **Mean**  **(Baseline)** | **Mean**  **(Retest)** | **Pearson *r*** | **ICC** |
| --- | --- | --- | --- | --- |
| 1 | 2.36 | 2.36 | **.34** | **.341** |
| 2 | 1.66 | 1.42 | **.54** | **.498** |
| 3 | 1.36 | 1.33 | **.56** | **.553** |
| 4 | 1.94 | 1.88 | **.65** | **.643** |
| 5 | 2.54 | 2.34 | **.37** | **.366** |
| 6 | 2.77 | 2.69 | **.55** | **.547** |
| 7 | 1.66 | 1.73 | **.43** | **.434** |
| 8 | 1.52 | 1.58 | **.52** | **.510** |
| 9 | 2.30 | 2.32 | **.31** | **.310** |
| 10 | 2.67 | 2.41 | **.28** | **.278** |
| 11 | 1.95 | 2.01 | **.37** | **.373** |
| 12 | 2.01 | 1.91 | **.32** | **.313** |
| 13 | 1.62 | 1.58 | **.41** | **.414** |
| 14 | 1.77 | 1.69 | **.40** | **.395** |
| 15 | 2.38 | 2.33 | **.45** | **.442** |
| 16 | 1.63 | 1.62 | **.28** | **.279** |
| 17 | 2.03 | 1.97 | **.36** | **.363** |
| 18 | 1.69 | 1.71 | **.28** | **.281** |
| 19 | 1.78 | 1.80 | **.31** | **.309** |
| 20 | 1.54 | 1.58 | **.26** | **.258** |
| 21 | 2.07 | 2.07 | **.41** | **.413** |
| 22 | 1.90 | 1.82 | **.32** | **.316** |
| 23 | 2.42 | 2.30 | **.25** | **.250** |
| 24 | 2.16 | 2.05 | **.24** | **.235** |
| 25 | 1.58 | 1.48 | **.33** | **.330** |
| 26 | 1.91 | 1.92 | **.26** | **.264** |
| 27 | 1.91 | 1.90 | **.42** | **.423** |
| 28 | 1.60 | 1.60 | **.46** | **.462** |
| 29 | 1.61 | 1.53 | **.23** | **.229** |
| 30 | 1.58 | 1.43 | **.25** | **.236** |

*Note*: Intraclass Correlation Coefficient (ICC) are two-way mixed-effects, absolute-agreement, single-measure estimates.
